# Inhibitory characteristics of flavonol-3-*O*-glycosides from *Polygonum aviculare* L. (common knotgrass) against porcine pancreatic lipase

**DOI:** 10.1038/s41598-019-54546-8

**Published:** 2019-12-02

**Authors:** Jun-Young Park, Chung Sun Kim, Kyung-Min Park, Pahn-Shick Chang

**Affiliations:** 1 Department of Agricultural Biotechnology, Seoul National University, Seoul, 08826 Republic of Korea; 2 Department of Food Science and Biotechnology, Wonkwang University, Iksan, 54538 Republic of Korea; 3 Center for Food and Bioconvergence, Seoul National University, Seoul, 08826 Republic of Korea; 4 Research Institute of Agriculture and Life Sciences, Seoul National University, Seoul, 08826 Republic of Korea

**Keywords:** Hydrolases, Biocatalysis

## Abstract

Pancreatic lipase (PL) is an enzyme that plays an essential role in the digestion of dietary lipids and is a suitable target for an anti-obesity dietary supplement. The objective of this study was to find a novel source of PL inhibitors from Korean medicinal plants and investigate the PL-inhibitory properties of the active constituents. From among 34 kinds of methanolic crude extracts, *Polygonum aviculare* L. showed the highest PL-inhibitory activity (63.97 ± 0.05% of inhibition). Solvent fractionation and liquid chromatography/mass spectrometry (LC/MS) analysis identified flavonol-3-*O*-glycosides, flavonol-3-*O*-(2″-galloyl)-glycosides, and flavonol aglycones as active constituents. Furthermore, the inhibitory characteristics of the major compounds were investigated in terms of enzyme kinetics and fluorescence quenching. The results suggested that the inhibitory activity of the major compounds is closely related to the tertiary structural change in PL, and that differences in inhibitory activity occurred due to slight discrepancies in their chemical structure.

## Introduction

Obesity, considered an ongoing public health issue in the 21^st^ century, is defined as excessive fat accumulation and superfluous body weight for height^1^. The global prevalence of obesity has increased steadily through the years since the first obesity survey in 1975 where, worldwide, 39% (>1.9 billion) of adults were overweight and 13% (>650 million) were obese in 2016; moreover, the trend has shown no clear sign of retrenchment^2,3^. More recently, particular attention has been paid to obesity in young people, because the number of obese children and adolescents has increased more than 10-fold in the past four decades^4^. Obesity is commonly accompanied by several comorbidities that are among the leading risk factors for death, such as cardiovascular disease, ischemic stroke, and type II diabetes mellitus^1^. Hence, finding a solution to the obesity epidemic is a public health priority^5^. The etiology of obesity is very complex; however, the major driver is an energy imbalance caused by overeating (*i*.*e*., high intake of energy-dense food) and a sedentary lifestyle^6^. Especially, excessive intake of lipids and carbohydrates beyond the energy needs of the individual can disrupt lipid metabolism and homeostasis, leading to increased fat deposition and blood lipid concentrations (*e*.*g*., triglyceride and low-density lipoprotein)^1,7,8^. In this context, the development of strategies to control lipid intake has been a major target in medical and dietary supplement research.

A variety of different enzymes involved in lipid metabolism are currently being identified and represent a rich pool of potential targets for obesity treatments^8^. Among these, pancreatic lipase (PL; EC 3.1.1.3), which plays an essential role in the digestion of dietary lipids (about 50–70% of total dietary lipids)^9^, is a primary modulator of lipid metabolism and thus can be the most suitable target for the anti-obesity agent. In other words, one of the best strategies for the treatment of obesity includes inhibition of PL (*i*.*e*., development of an inhibitor), with the goal of reducing the absorption of dietary lipids through gastrointestinal mechanisms without altering any central mechanisms^9^. Orlistat (also known as tetrahydrolipstatin), which is a notable example of an anti-obesity drug approved by the US FDA, specifically inhibits PL and prevents the hydrolysis and absorption of dietary lipids; however, safety issues have arisen because of its severe side effects including fatty and oily stool, fecal urgency, oily spotting, and flatulence^8,10^. Therefore, the development of safety-ensured inhibitors has become a priority, and natural sources have emerged as a major area of interest with respect to anti-obesity dietary supplements. In particular, numerous studies on plant extracts have contributed to the identification of various potent inhibitors of PL^9,11,12^. On the contrary to this, there is insufficient research on the PL-inhibitory activity of Korean medicinal plants and their active constituents, although they have been eaten traditionally to maintain wellness in Korea. Accordingly, we hypothesized that Korean medicinal plants could serve as novel sources of PL inhibitors for use in anti-obesity agents.

In this study, we assessed the PL-inhibitory activities of 34 kinds of Korean medicinal plants closely related to the obesity and selected *Polygonum aviculare* L. (also known as common knotgrass) as a novel source of PL inhibitors. *P. aviculare* (a member of the family Polygonaceae), which is widespread in Korea and also in the Occident, has traditionally been used as an esculent plant for diuretic, astringent, and antihypertensive purposes^13^. However, there are few studies on the anti-obesity functionality of *P. aviculare*, especially its inhibitory activity against PL^14,15^. Therefore, the primary objective of this study was to investigate the relevant properties of *P. aviculare* and assess its potential as a novel source of PL inhibitors. We successfully identified the PL-inhibitory constituents in the solvent fractions of *P. aviculare* by liquid chromatography/mass spectrometry (LC/MS) analysis and evaluated the inhibitory characteristics of its major compounds by enzyme kinetics and fluorescence analysis.

## Results and Discussion

### Screening for PL-inhibitory activities of Korean plants

Crude extracts of 34 kinds of Korean medicinal plants were prepared using 80%(v/v) aqueous methanol, which can dissolve both unknown hydrophilic and hydrophobic compounds effectively. The PL-inhibitory activities of these crude extracts were evaluated at the same concentration (1.25 mg/mL), and the results are given in Table 1. Most of the crude extracts (1−30) had PL-inhibitory activity to various extents, and this phenomenon was explained by previous reports on natural PL-inhibitory compounds in plants, such as polyphenols, saponins, and terpenes^9^. The PL reactions reached equilibrium very rapidly even though crude extracts inhibited the reactions (see Supplementary Fig. S1), which means that the putative inhibitor compounds would show a conventional mechanism of inhibition (*i*.*e*., fast reversible inhibition). Consequently, *P. aviculare* showed the significantly highest (*p* < 0.05) PL inhibition (63.97 ± 0.05%) among any other plant species. *P. aviculare* has not been mentioned previously as a source of PL inhibitors; hence, it was chosen as the final target for study and analyzed to identify its major compounds.

**Table 1:** Pancreatic lipase inhibitory activities of crude extracts from Korean medicinal plants.

No.	Species	Family	Inhibition (%)
1	*Polygonum aviculare* L.	Polygonaceae	63.97 ± 0.05^a^
2	*Sigesbeckia glabrescens* (Makino) Makino	Compositae	59.94 ± 0.01^b^
3	*Senna tora* (L.) Roxb.	Leguminosae	57.87 ± 0.60^c^
4	*Phyllostachys nigra* var. *henonis* (Bean) Stapf ex Rendle	Gramineae	57.59 ± 0.41^c^
5	*Epimedium koreanum* Nakai	Berberidaceae	57.32 ± 2.03^c^
6	*Prunella vulgaris* var. *lilacina* Nakai	Labiatae	56.88 ± 0.85^c^
7	*Taxus cuspidata* Siebold & Zucc.	Taxaceae	54.85 ± 0.31^d^
8	*Dendranthema indicum* (L.) Des Moul.	Compositae	54.34 ± 0.02^d^
9	*Pueraria lobata* (Willd.) Ohwi	Leguminosae	53.74 ± 1.36^d^
10	*Artemisia annua* L.	Compositae	52.80 ± 0.07^d^
11	*Trachelospermum asiaticum* (Siebold & Zucc.) Nakai	Apocynaceae	46.32 ± 0.10^e^
12	*Crataegus pinnatifida* Bunge	Rosaceae	46.27 ± 0.58^e^
13	*Morus alba* L.	Moraceae	45.72 ± 0.89^e^
14	*Schisandra chinensis* (Turcz.) Baill.	Schisandraceae	42.14 ± 1.56 ^f^
15	*Equisetum hyemale* L.	Equisetaceae	39.46 ± 1.21 ^g^
16	*Cirsium japonicum* var. *maackii* (Maxim.) Matsum.	Compositae	37.59 ± 0.67 ^g^
17	*Equisetum arvense* L.	Equisetaceae	32.61 ± 1.33 ^h^
18	*Glycyrrhiza uralensis* Fisch.	Leguminosae	32.60 ± 1.55 ^h^
19	*Aralia cordata* var. *continentalis* (Kitag.) Y.C.Chu	Araliaceae	29.97 ± 1.50^i^
20	*Cnidium officinale* Makino	Umbelliferae	26.89 ± 1.39^j^
21	*Zea mays* L.	Gramineae	23.93 ± 1.07^k^
22	*Viscum album* var. *coloratum* (Kom.) Ohwi	Loranthaceae	20.95 ± 0.41 ^l^
23	*Lycium chinense* Mill.	Solanaceae	17.31 ± 0.84 ^m^
24	*Trichosanthes kirilowii* Maxim.	Cucurbitaceae	13.86 ± 0.10^n^
25	*Aralia elata* (Miq.) Seem.	Araliaceae	13.66 ± 0.95^n^
26	*Rehmannia glutinosa* (Gaertn.) Libosch. ex Steud.	Scrophulariaceae	13.58 ± 0.95^n^
27	*Aconitum pseudolaeve* Nakai	Ranunculaceae	10.97 ± 1.10^o^
28	*Eucommia ulmoides* Oliv.	Eucommiaceae	10.55 ± 1.15^o^
29	*Allium tuberosum* Rottler ex Spreng.	Liliaceae	6.69 ± 1.24^p^
30	*Scrophularia buergeriana* Miq.	Scrophulariaceae	3.62 ± 0.52^q^
31	*Lithospermum erythrorhizon* Siebold & Zucc.	Boraginaceae	−3.44 ± 0.76^r^
32	*Solanum lyratum* Thunb.	Solanaceae	−14.45 ± 3.90^s^
33	*Codonopsis pilosula* (Franch.) Nannf.	Campanulaceae	−19.75 ± 1.04^t^
34	*Liriope platyphylla* F.T. Wang & T. Tang	Liliaceae	—

^a–t^Different superscripts represent statistically significant differences (*p* < 0.05) between mean values of the magnitude of inhibition analyzed by Duncan’s multiple range test on ANOVA.

**Figure 1:**
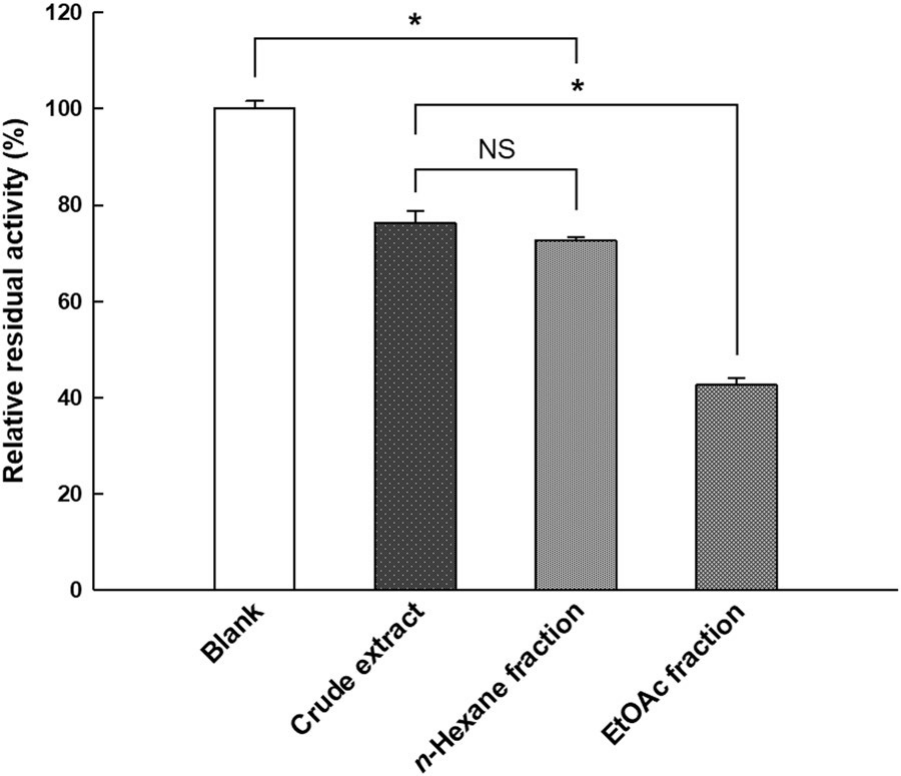
Comparison of the pancreatic lipase (PL)-inhibitory activities of *Polygonum aviculare* L. crude extract and its *n*-hexane/ethyl acetate (EtOAc) fractions. The concentration of all samples was 0.25 mg/mL. The PL-inhibitory activity of the EtOAc fraction was significantly stronger than that of the other fractions. Error bars indicate standard error of the mean. Asterisks between bars denote significant differences in PL-inhibitory activity (*p* < 0.01, Duncan’s multiple range test on ANOVA). NS, not significantly different (*p* > 0.01).

The methanolic crude extract of *P. aviculare* was successively fractionated with *n*-hexane and EtOAc solvents in polarity order. After every solvent fractionation, we were able to obtain about 0.5−1.0 mg of lyophilized *n*-hexane and EtOAc fractions of *P. aviculare* uniformly. The relative residual activity (RRA) of PL in the presence of 0.25 mg/mL crude extract and *n*-hexane fraction was 76.30 ± 2.47% and 72.69 ± 0.66%, respectively, as shown in Fig. 1. Because there was no significant difference between these (*p* > 0.01), it was considered that inherent active constituents in the crude extract were not specifically fractionated by *n*-hexane with a high level of relative polarity (0.009)^16^. On the other hand, the EtOAc fraction exhibited significantly higher PL-inhibitory activity (42.62 ± 1.49% of RRA) than those of the crude extract and *n*-hexane fraction (*p* < 0.01), which means that EtOAc with a moderate level of polarity (0.228) was able to fractionate a group of compounds including the active constituents required for PL inhibition^16^. According to previous studies, *P. aviculare* commonly contains many phenolic compounds, such as flavonoids and flavonoid glycosides^17,18^. As these compounds can be fractionated by EtOAc and may have PL-inhibitory activities, we expected that flavonoid compounds in *P. aviculare* could be major compounds in PL inhibition^12^.

### Chemical identification of EtOAc fraction by UPLC-ESI-MS analysis

**Figure 2:**
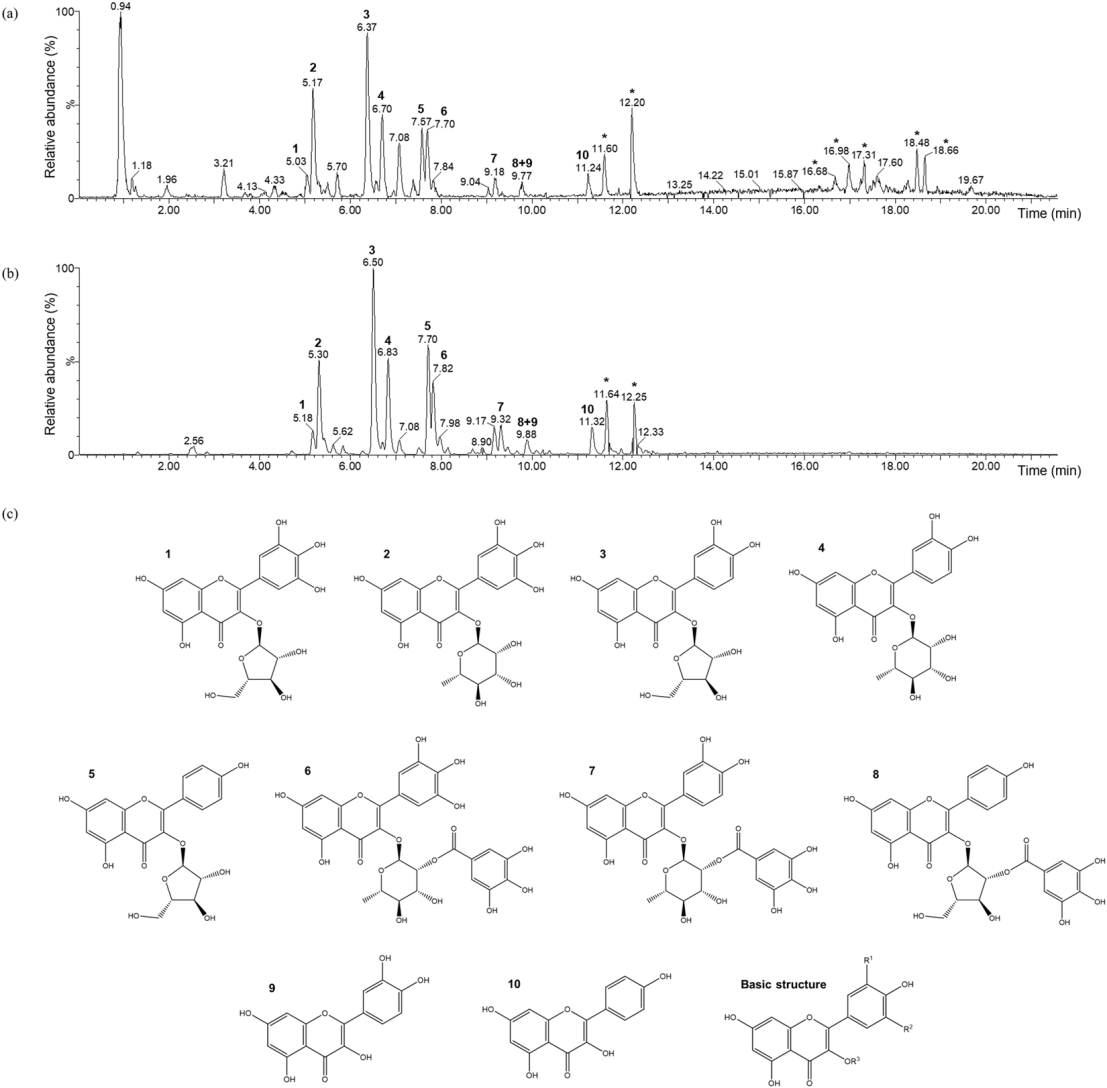
Ultra-performance liquid chromatography coupled with electrospray ionization mass spectrometry (UPLC-ESI-MS) analysis of *Polygonum aviculare* L. crude extract and its ethyl acetate (EtOAc) fraction. (**a**) UPLC chromatogram of crude extract. (**b**) UPLC chromatogram of EtOAc fraction. (**c**) Chemical structures of annotated compounds in the EtOAc fraction. The EtOAc fraction was mostly composed of 10 kinds of flavonoids (1–10) and also contained several fatty acids (*).

We performed UPLC-ESI-MS analysis of the crude extract and EtOAc fraction of *P. aviculare* to identify the EtOAc-fractionated compounds. The UPLC chromatograms of each sample are shown in Fig. 2. The methanolic crude extract was a mixture of hydrophilic and hydrophobic compounds (see Fig. 2a), whereas the EtOAc fraction only contained compounds with the moderate polarity expected in a family of flavonoids (see Fig. 2b). All distinguishable peaks in the chromatogram were successively analyzed by ESI-QTOF MS, which is suitable for natural compound analysis, and the results were compared with previous reports on the *P. aviculare*^17–19^. Consequently, most of the compounds in the EtOAc fraction were annotated to flavonoids, as expected. Structural information of 10 compounds with a high relative abundance in the EtOAc fraction is provided in Fig. 2c and Table 2. Flavonoids in the EtOAc fraction consisted of flavonol-3-*O*-glycosides (1−5), flavonol-3-*O*-(2″-galloyl)-glycosides (6−8), and flavonol aglycones (9−10). The largest proportion was flavonol-3-*O*-glycosides of kaempferol, quercetin, and myricetin with rhamnose or arabinose at the position of the C-3 hydroxyl group; hence, avicularin (quercetin-3-*O*-α-L-arabinofuranoside), myricitrin (myricetin-3-*O*-α-L-rhamnopyranoside), and quercitrin (quercetin-3-*O*-α-L-rhamnopyranoside) were the major compounds in PL inhibition by *P. aviculare*. Several weak peaks in the chromatogram require further fractionation to identify all constituents clearly; however, the MS results of major peaks, which probably exert the largest influence on the PL-inhibitory activity, were highly reliable because there is no large discrepancy (below 5 ppm) between the calculated and observed molecular weight for all constituents.

**Table 2:** Compounds annotated by mass spectrometry analysis in the ethyl acetate fraction of *Polygonum aviculare* L.

No.	Compound	Retention time (min)	Observed m/z [M-H]^−^	Molecular weight	Molecular formula	R^1^	R^2^	R^3^
1	Betmidin	5.18	449.073	450.352	C_20_H_18_O_12_	OH	OH	α-L-arabinofuranosyl
2	Myricitrin	5.30	463.087	464.379	C_21_H_20_O_12_	OH	OH	α-L-rhamnopyranosyl
3	Avicularin	6.50	433.077	434.353	C_20_H_18_O_11_	OH	H	α-L-arabinofuranosyl
4	Quercitrin	6.83	447.093	448.380	C_21_H_20_O_11_	OH	H	α-L-rhamnopyranosyl
5	Juglanin	7.70	417.082	418.354	C_20_H_18_O_10_	H	H	α-L-arabinofuranosyl
6	Desmanthin-1	7.82	615.098	616.484	C_28_H_24_O_16_	OH	OH	(2″-*O*-galloyl)-*O*-α-L-rhamnopyranosyl
7	Quercetin-3-*O*-α-L-(2″-galloyl)-rhamnopyranoside	9.32	599.104	600.481	C_28_H_24_O_15_	OH	H	(2″-*O*-galloyl)-*O*-α-L-rhamnopyranosyl
8	Kaempferol-3-*O*-α-L-(2″-galloyl)-arabinofuranoside	9.88	569.094	570.455	C_27_H_22_O_14_	H	H	(2″-*O*-galloyl)-*O*-α-L-arabinofuranosyl
9	Quercetin	9.88	301.035	302.238	C_15_H_10_O_7_	OH	H	H
10	Kaempferol	11.32	285.039	286.239	C_15_H_10_O_6_	H	H	H

R^1^, R^2^, and R^3^ are substituent positions on the basic structure of flavonol in Fig. 2.

Flavonols are a class of flavonoids that have the 3-hydroxyflavone backbone, and their diversity stems from the different positions of the phenolic hydroxyl groups^20^. As several flavonols and flavonol glycosides have been reported to have PL-inhibitory activity, the same activity of *P. aviculare* in this study could be explained by its inherent flavonol glycosides^12^. The majority of compounds in the EtOAc fraction of *P. aviculare* were largely unknown about their PL-inhibitory activities; therefore, *P. aviculare* could be proposed as a novel source of PL inhibitors for anti-obesity agents, based on the inhibitory characteristics of its major compounds. In addition, we checked the total flavonoid contents of the crude extract and EtOAc fraction of *P. aviculare*. The contents of flavonoids in the crude extract and EtOAc fraction were 27.66 ± 0.72 µg of quercetin equivalent/mg of crude extract and 226.46 ± 4.15 µg of quercetin equivalent/mg of EtOAc fraction, respectively. The total flavonoid contents increased about 8-fold after EtOAc fractionation. This implies that a high content of flavonoids could contribute to PL-inhibitory activity, thus paralleling the results of the UPLC-ESI-MS analysis.

### Inhibition kinetics of promising candidates for PL inhibitors

**Figure 3:**
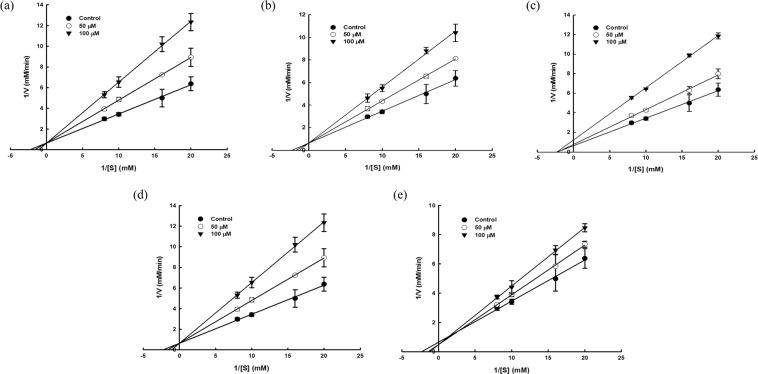
Lineweaver-Burk plots for the determination of inhibition type of the major compounds and flavonol aglycones against pancreatic lipase. (**a**) Quercetin. (**b**) Kaempferol. (**c**) Myricitrin. (**d**) Quercitrin. (**e**) Avicularin. The experiments were conducted under the concentrations of compounds at 50 μM and 100 μM together with blank.

The PL-inhibitory activity of major compounds (avicularin, myricitrin, and quercitrin) and flavonol aglycones (kaempferol, quercetin) in the EtOAc fraction of *P. aviculare* were converted into the half-maximal inhibitory concentration (IC_50_) by enzyme kinetics analysis. The IC_50_ is a practical measure of the potency of a compound for inhibiting a specific enzyme, and facilitates quantitative comparison of the inhibitory activity of different compounds. As shown in Table 3, the IC_50_ values of quercetin, kaempferol, myricitrin, quercitrin, and avicularin against PL were 53.05, 79.38, 92.85, 100.56, and 141.84 µM, respectively. These values were calculated from the RRA of PL in the presence of the compounds at various concentrations (Supplementary Fig. S2). The PL-inhibitory activities of quercetin, kaempferol, and quercitrin had been reported previously^12,21^; however, those of myricitrin and avicularin were reported for the first time in this study. Furthermore, the inhibition modes of each compound were elucidated by evaluating the changes of the kinetic parameters from double-reciprocal Lineweaver-Burk plots under the presence of 50 μM and 100 μM of each compound (Fig. 3). Myricitrin, quercitrin, and avicularin which belong to flavonol-3-*O*-glycosides showed non-competitive or mixed-type inhibition, whereas flavonol aglycones (quercetin and kaempferol) showed competitive inhibition.

**Table 3:** Half-maximal inhibitory concentrations (IC_50_) and fluorescence quenching parameters of major compounds and flavonol aglycones.

Compound	IC_50_ (μM)	Temperature (°C)	*K*_SV_ (10^4^ M^−1^)*	*k*_q_ (10^12^ M^−1^s^−1^)^**^	*K*_A_ (10^4^ M^−1^)^***^	n^****^	R^1^	R^2^	R^3^
Quercetin	53.05	25	1.00	6.29	—	—	OH	H	H
		37	0.94	5.91	3.57	1.1471			
Kaempferol	79.38	25	1.22	7.67	—	—	H	H	H
		37	1.03	6.48	2.07	1.1103			
Myricitrin	92.85	25	1.19	7.48	—	—	OH	OH	α-L-rhamnopyranosyl
		37	1.23	7.74	1.45	1.0228			
Quercitrin	100.56	25	1.28	8.05	—	—	OH	H	α-L-rhamnopyranosyl
		37	1.32	8.30	1.00	0.9760			
Avicularin	141.84	25	0.56	3.52	—	—	OH	H	α-L-arabinofuranosyl
		37	0.59	3.71	0.94	1.1119			

R^1^, R^2^, and R^3^ are substituent positions on the basic structure of flavonol in Fig. 2.

^*^Stern-Volmer quenching constant; ^**^Bimolecular quenching constant; ^***^Binding constant; ^****^Number of binding sites.

Collectively, it was found that these compounds showed quite different PL-inhibitory characteristics despite their similar structures (see Fig. 2c). These results indicate that differences in inhibitory activity occurred according to slight discrepancies in structure, such as hydroxylation in B-ring of flavone backbone, the presence of glycosylation at the position of C-3 hydroxyl group, and the type of glycosidic sugar. Especially, quercetin and kaempferol exhibited higher inhibitory activity than myricitrin, quercitrin, and avicularin. Glycosylation can strongly influence the hydrophilicity and steric hindrance of a structure^22^, affecting the interaction between enzyme and inhibitor (*i*.*e*., inhibitory activity). Therefore, the presence of rhamnopyranose or arabinofuranose in major compounds rendered their structures inaccessible to PL and lowered the inhibitory activity. Similar to our studies, the glycosylation of flavonoids has been shown to cause a decrease in PL-inhibitory activity in previous studies^21^. In addition, an influence of hydroxylation and glycosidic sugar type on inhibitory activity can be inferred from our data. For the future, experiments focusing on addressing the comparative analysis and in-depth structural analysis to reveal these effects would make explicit the findings from this study.

### Molecular interaction between PL and promising candidates for PL inhibitors

**Figure 4:**
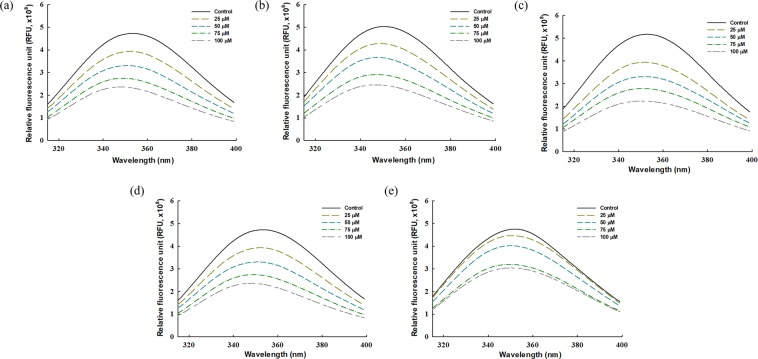
Fluorescence emission spectra of pancreatic lipase in the presence of the major compounds and flavonol aglycones. (**a**) Quercetin. (**b**) Kaempferol. (**c**) Myricitrin. (**d**) Quercitrin. (**e**) Avicularin. The solid line shows the fluorescence emission spectra in the absence of compounds.

In most cases, the inhibitory activity cannot demonstrate all changes in an enzyme caused by an inhibitor. To understand the interaction between PL and the major compounds in the EtOAc fraction of *P. aviculare*, fluorescence analysis of PL was performed in the absence and presence of the major compounds and flavonol aglycones. First, the fluorescence emission spectra (excitation at 295 nm) of PL in the presence of each compound were obtained, as shown in Fig. 4. The fluorescence emission intensity of PL decreased with an increase in the concentration of the inhibitory compound, and a slight blue shift (*i*.*e*., a decrease in wavelength) was observed in the emission maximum wavelength (2−6 nm). This phenomenon is explained by fluorescence quenching of tryptophan in protein; in other words, the inhibitory compound interacted with PL and the consequent changes in PL structure quenched the intrinsic fluorescence of tryptophan in PL. The anisotropy displayed by tryptophan in protein is sensitive to both the overall rotational diffusion of proteins and the extent of segmental motion during the excited state; for that reason, the intrinsic fluorescence of tryptophan (selectively excited at 295−305 nm) in protein can provide considerable information about protein structure and dynamics^23^. Most importantly, we found that greater fluorescence quenching (especially blue shift) occurred when the IC_50_ value of the inhibitory compound was smaller, which implies that the PL-inhibitory activity of the compounds is closely related to the structural change in PL, in particular the tertiary structure.

Second, we derived several parameters (*K*_SV_, *k*_q_, *K*_A_, and *n*) of fluorescence quenching (see Table 3) from the Stern-Volmer equation (see Supplementary Fig. S3) and its double-logarithmic equation (see Supplementary Fig. S4). The *K*_SV_ values at 37 °C were not significantly different from the *K*_SV_ values at 25 °C, probably due to the strong formation of the protein-quencher complex. On the other hand, the calculated *k*_q_ values at both temperatures were higher than the maximum diffusion collision quenching rate constant (10^10^ M^−1^s^−1^). This status can be defined as static quenching, which means that fluorescence quenching was caused by the formation of a complex between protein and molecule^23^. Taken together, the results of the fluorescence analysis suggest that flavonol glycosides and flavonol aglycones in the EtOAc fraction of *P. aviculare* manifest their PL-inhibitory activities by interacting with PL, thereby forming the complex and ultimately modifying the tertiary structure. Our suggestion corresponds with previous studies on enzyme inhibition by flavonoid compounds, which proposed the formation of a complex between the enzyme and inhibitor as the mode of action^24,25^.

Finally, Table 3 also presents the *K*_A_ and *n* values of each compound obtained with the double-logarithmic equation. The number of binding sites (*n*) of each compound was close to one, indicating that only one binding site existed in the PL. The binding constants (*K*_A_) for quercetin, kaempferol, myricitrin, quercitrin, and avicularin were 3.57 × 10^4^, 2.07 × 10^4^, 1.45 × 10^4^, 1.00 × 10^4^, and 0.94 × 10^4^ M^−1^, respectively. The *K*_A_ value indicates the ability to combine with protein, and thus may be closely related to inhibitory activity. As expected, the relation between these *K*_A_ values and the IC_50_ values corroborated our suggestion regarding the PL-inhibitory characteristics of flavonol glycosides and flavonol aglycones, because the order of the *K*_A_ value was contrary to the order of the IC_50_ value (*i*.*e*., quercetin > kaempferol > myricitrin > quercitrin > avicularin for PL-inhibitory activity). In addition, CD analysis was performed to understand the effect of the major compounds on the secondary structure of PL. As shown in Table 4, the composition of secondary structures of PL was as follows: α-helix (18.7%), antiparallel β-sheet (33.0%), parallel β-sheet (12.8%), β-turn (20.0%), and random coil (43.8%). However, no significant discrepancy was found in the secondary structure of PL, compared with the results of other studies^24–26^. Hence, the effect of the major compounds on the secondary structure of PL was negligible.

**Table 4:** Conformational changes in the secondary structure of pancreatic lipase in the presence of each major compounds and flavonol aglycones.

	Control (%)	Quercetin		Kaempferol		Myricitrin		Quercitrin		Avicularin	
		200 (μM)	400	200	400	200	400	200	400	200	400
α-Helix	18.7	−0.4	−0.3	+ 0.0	−0.2	−0.4	−0.5	−0.2	−0.2	−0.1	−0.1
Antiparallel β-sheet	33.0	+ 1.6	+ 0.8	−0.5	+ 0.6	+ 1.0	+ 0.6	+ 0.0	+ 0.0	−0.1	−0.2
Parallel β-sheet	12.8	+ 0.1	+ 0.2	+ 0.1	+ 0.0	+ 0.2	+ 0.3	+ 0.2	+ 0.2	+ 0.1	+ 0.1
β-Turn	20.0	+ 0.1	+ 0.0	−0.1	+ 0.0	+ 0.0	+ 0.0	−0.1	−0.1	−0.1	−0.1
Random coil	43.8	+ 0.3	+ 0.8	+ 0.6	+ 0.2	+ 0.8	+ 1.3	+ 0.8	+ 0.8	+ 0.4	+ 0.6

In conclusion, we have proposed *P. aviculare* as a novel source of PL inhibitors. The EtOAc fraction of *P. aviculare* exerted high PL-inhibitory activity and it was composed of flavonol-3-*O*-glycosides, flavonol-3-*O*-(2″-galloyl)-glycosides, and flavonol aglycones. Furthermore, inhibitory characteristics of major compounds in *P. aviculare* were investigated by enzyme kinetics and fluorescence quenching analysis. The scope of this study was limited in terms of *in vitro* PL inhibition; however, the findings have a number of important implications for future practice, such as *in vivo* PL inhibition and anti-obesity functionality. Therefore, as well as demonstrating a series of scientific processes for identifying a novel source of PL-inhibitors from natural sources, our results could provide information aiding effective utilization of *P. aviculare* as an anti-obesity agent based on PL inhibition. Additionally, since the plant *P*. *aviculare* are quite affordable in Korea as well as across many countries in temperate regions of Eurasia and North America, it would be an excellent material for practical utilization.

## Methods

### Materials

All plant samples were collected from Goesan-gun (Chungcheongbuk-do, Republic of Korea) in 2015 and were immediately cleaned, ground, lyophilized, and stored at −20 °C before use. Lipase from porcine pancreas type II, 4-methylumbelliferyl oleate (4-MUO), and quercetin (≥95%) was purchased from Sigma-Aldrich Co. (St. Louis, MO, USA). Kaempferol (≥98.3%), myricitrin (≥98.3%), quercitrin (≥98.5%), and avicularin (≥99.3%) were purchased from ChemFaces Biochemical Co. (Wuhan, Hubei, China), and their purity was confirmed analytically by proton nuclear magnetic resonance (^1^H-NMR) and HPLC. Methanol, *n*-hexane, and ethyl acetate (EtOAc) were purchased from Daejung Chemicals & Metals Co. (Siheung-si, Gyeonggi-do, Republic of Korea), and were of analytical grade. Water and acetonitrile purchased from J. T. Baker Co. (Phillipsburg, NJ, USA) were of high-performance liquid chromatography (HPLC) grade. The acetonitrile was dehydrated by molecular sieves 4 Å (Sigma-Aldrich Co.) and filtered through a membrane filter (0.45 µm) before use. All of the other chemicals were of extra-pure grade.

### Solvent extraction and fractionation

The Korean plant sample (10 g) was homogenized with 200 mL of 80%(v/v) aqueous methanol at room temperature for 24 h and then filtered through a 0.5 µm polytetrafluoroethylene (PTFE) membrane filter (Advantec MFS Co., Tokyo, Japan). The filtrate was concentrated by an EYELA N-1100 rotary evaporator (Tokyo Rikakikai Co., Tokyo, Japan), and the concentrate was lyophilized by an FD 8512 freeze dryer (IlShin BioBase Co., Gyeonggi-do, Republic of Korea) at −76 °C to obtain a crude methanol extract of the plant sample. The obtained crude extract (7 g) was fractionated by suspending it in water (200 mL) and *n*-hexane (200 mL) for 2 h, and the *n*-hexane layer was separated using a separatory funnel. The EtOAc layer was successively separated from the aqueous layer using the same method. The *n*-hexane and EtOAc layers were lyophilized to obtain each solid fraction.

### Determination of total flavonoid content

The total flavonoid contents of the crude extract and the EtOAc fraction were determined by the aluminum chloride (AlCl_3_) colorimetric method with a slight modification^27^. A volume of 0.5 mL of the sample was mixed with 0.1 mL of 10%(w/v) AlCl_3_ solution, 0.1 mL of 0.1 mM potassium acetate, 1.5 mL of methanol, and 2.8 mL of distilled water. After incubation of 30 min, the absorbance of the mixture was measured at 415 nm with a UV-2450 ultraviolet-visible spectrophotometer (Shimadzu Co., Kyoto, Japan). The total flavonoid content was expressed as µg quercetin equivalents per mg dry weight of the sample, and the standard curve is shown in Supplementary Fig. S5. All prepared solutions were filtered through a membrane filter (0.45 µm).

### UPLC-ESI-MS analysis

Ultra-performance liquid chromatography (UPLC) coupled with electrospray ionization mass spectrometry (ESI-MS) was used to identify the constituents in the crude extract and EtOAc fraction. UPLC was performed using an Acquity UPLC system (Waters Co., Manchester, UK) consisting of a binary solvent delivery system and an autosampler. The samples were separated by a Waters Acquity UPLC BEH C_18_ column (2.1 mm × 100 mm, 1.7 µm) and introduced into a Waters Xevo G2 QTOF (quadrupole time-of-flight) MS through an ESI interface. The mobile phase was composed of 0.1%(v/v) formic acid in water (A) and acetonitrile (B), using linear gradient elution with 10−25% B at 0−8 min, 25−85% at 8−20 min, and 85−100% at 20−23 min. The injection volume was 2.0 µL and the flow rate was maintained at 0.3 mL/min at all times. The temperatures in the autosampler and the column oven were set at 15 °C and 40 °C, respectively. The UPLC effluent entered the MS at a flow rate of 0.3 mL/min through an electrospray source with the capillary set at 3.0 kV, a source block temperature of 120 °C, and a desolvation gas temperature of 350 °C. Nitrogen was used as both cone gas and desolvation gas at flow rates of 50 and 800 L/h, respectively. The samples were ionized in the negative ion mode and their MS data were acquired.

### Pancreatic lipase inhibition assay

The PL assay using 4-MUO as a substrate was conducted as described previously with slight modification^28,29^. Lipase from porcine pancreas type II (*i*.*e*., pancreatic lipase) was dissolved in 50 mM Tris-HCl buffer (pH 8.0) to give 10 mg/mL, and centrifuged at 5,000 g for 10 min to remove insoluble material before the enzyme assay. Then, 50 µL of 0.5 mM 4-MUO solution dissolved in the above buffer and 100 µL of the sample solution dissolved in methanol (*i*.*e*., the inhibitor solution) were mixed in 96-well microplate to form the reaction system. The enzymatic hydrolysis was initiated by adding 50 µL of the PL solution to the reaction system (final 250 units/mL) at 37 °C. Fluorescence of 4-methylumbelliferone (4-MU), which is a product of hydrolysis of 4-MUO, was detected at an excitation wavelength of 320 nm and an emission wavelength of 455 nm using a SpectraMax^®^ i3 Multi-Mode Microplate Reader (Molecular Devices, San Jose, CA, USA). The inhibitory activities against PL were calculated for comparison with each other, as follows:

1 $$Inhibition\,( \% )=\frac{{A}_{0}-{A}_{S}}{{A}_{0}}\times 100$$

where *A*_0_ is the relative fluorescence unit after the reaction for 30 min in the absence of sample (*i*.*e*., only methanol) and *A*_*S*_ is the fluorescence unit in the presence of sample.

To determine the IC_50_, the RRA of PL in the presence of the sample at various concentrations was evaluated. The reaction of PL was measured by the same procedure described above, and the RRA was calculated from the following equation:

2 $$Relative\,residual\,activity\,( \% )=\frac{{v}_{S}}{{v}_{0}}\times 100$$

where *ν*_0_ is the initial velocity of the reaction in the absence of sample (*i*.*e*., only methanol) and *ν*_*S*_ is the initial velocity in the presence of sample. Nonlinear regression curve fitting of RRA against sample concentration was performed using the SigmaPlot software (ver. 12.5; Systat Software Co., CA, USA) and the IC_50_ was determined. The type of inhibition was elucidated by evaluating the changes of kinetic parameters from Lineweaver-Burk plot as follows:

3 $$\frac{1}{v}=\frac{{K}_{m}}{{V}_{max}}\frac{1}{[S]}+\frac{1}{{V}_{max}}$$

where [*S*] is the concentration of substrate, *v* is the initial velocity of the reaction, V_max_ is the maximum initial velocity when substrate approaches infinite concentration, and K_m_ is the dissociation constant of the enzyme-substrate complex (*i*.*e*., the Michaelis constant).

### Fluorescence emission spectroscopy

The fluorescence spectra of PL in the presence of the sample were measured on a SpectraMax^®^ i3 Multi-Mode Microplate Reader (Molecular Devices) as described previously with slight modification^30^. In brief, aliquots of the sample solution at various concentrations (0−100 µM) were added into 40 µM of PL solution. The mixture was incubated for 30 min at 25 °C and 37 °C, and the fluorescence emission spectra were measured in the range of 315−400 nm upon excitation at 295 nm. The excitation and emission slit widths were set at 9 and 15 nm, respectively. The fluorescence data were analyzed by fitting to the Stern-Volmer equation for analysis of fluorescence quenching^23^:

4 $$\frac{{F}_{0}}{F}=1+{K}_{{\rm{SV}}}[Q]=1+{k}_{{\rm{q}}}{\tau }_{0}[Q]$$

where *F*_0_ and *F* is the fluorescence intensity in the absence and presence of quencher, respectively, *k*_q_ is the bimolecular quenching constant, *τ*_0_ is the lifetime of fluorescence in the absence of quencher, *K*_SV_ is the Stern-Volmer quenching constant, and [*Q*] is the concentration of quencher. *K*_SV_ was obtained from the slope of a plot of *F*_0_/*F* versus [*Q*] by linear regression curve fitting, and *k*_q_ was calculated by *K*_SV_, where *τ*_0_ is equal to 1.59 ns^31^. The binding constant (*K*_A_) and number of binding sites (*n*) were obtained according to a double-logarithmic equation^32^:

5 $$\log \,\frac{{F}_{0}-F}{F}=\,\log \,{K}_{{\rm{A}}}+n\,\log \,[Q]$$

### Circular dichroism

The circular dichroism spectra of PL were detected by a Chirascan^TM^-plus CD spectrometer (Applied Photophysics Co., Surrey, UK) in the far-UV region (190−260 nm). The concentration of PL was 1.209 mg/mL, and the PL sample was transferred to a quartz cuvette with a path length of 0.2 mm. The resolution was 0.5 nm, time per point was 0.2 s, and bandwidth was 0.5 nm. The proportion of secondary structures of PL was calculated with Circular Dichroism analysis using Neural Networks (CDNN) software (http://gerald-boehm.de/index.php) in the absence and presence of sample.

### Statistical analysis

Statistical analysis was performed using SPSS statistics software (ver. 23.0; IBM Co., Armonk, NY, USA). All experiments were conducted in triplicate. The data were subjected to one-way analysis of variance (ANOVA), and significant differences among mean values were compared using Duncan’s multiple range test.

## Supplementary information

Supplementary Data (S1-S5)
