# Supplementary material for: Inhibitory characteristics of flavonol-3-O-glycosides from Polygonum aviculare L. (common knotgrass) against porcine pancreatic lipase
Source: Sci Rep. 2019 Dec 2;9:18080. doi: 10.1038/s41598-019-54546-8 (PMC6889161; doi:10.1038/s41598-019-54546-8)
Supplement: Supplementary file 1 — Supplementary Data (S1-S5) [file 41598_2019_54546_MOESM1_ESM.docx]

**Inhibitory characteristics of flavonol-3-*O*-glycosides from *Polygonum aviculare* L. (common knotgrass) against porcine pancreatic lipase**

Jun-Young Park, Chung Sun Kim, Kyung-Min Park, and Pahn-Shick Chang^*^

This file contains information about the supplementary data (S1-S5).


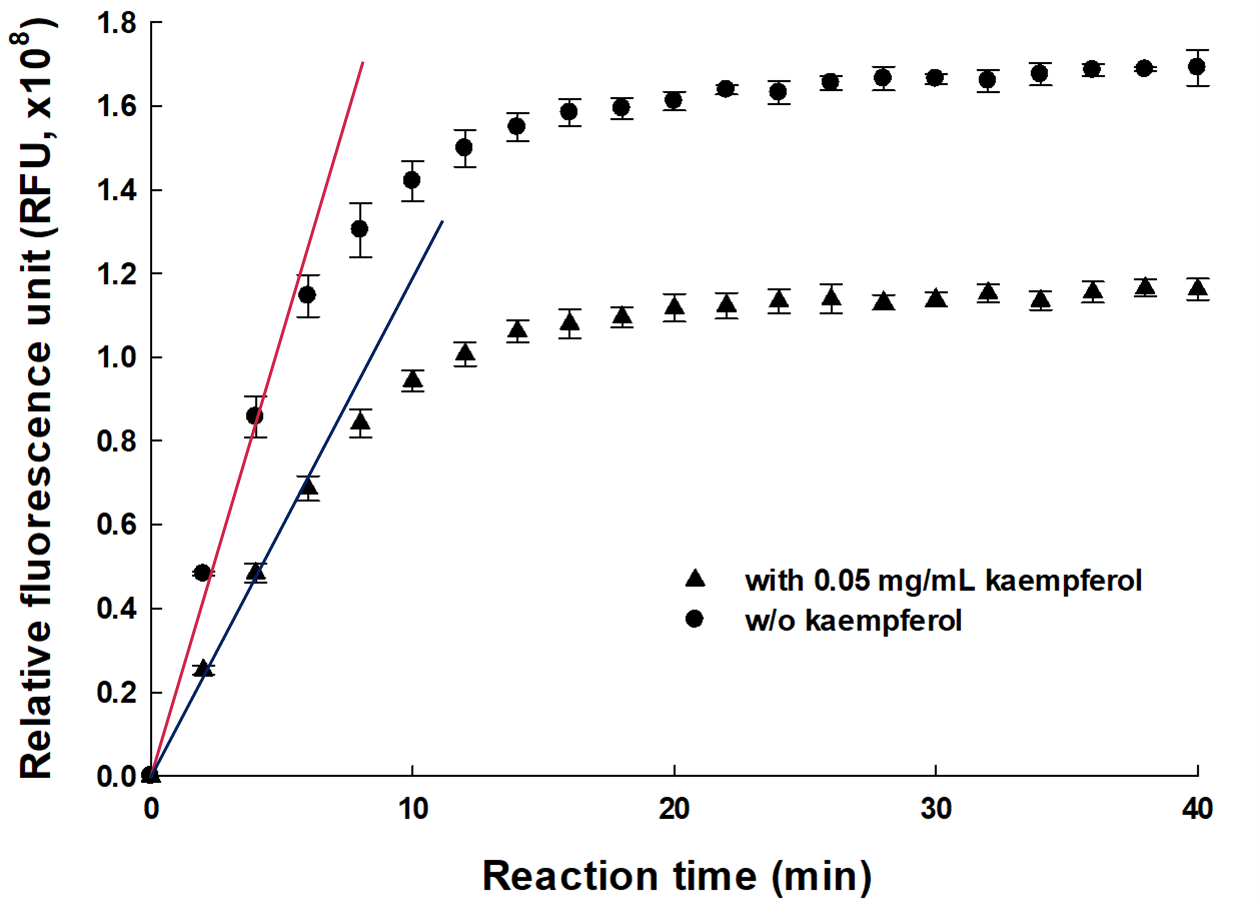


Figure S1. **Reaction of pancreatic lipase in the absence (●) and presence (▲) of 0.05 mg/mL kaempferol based on fluorescence assay.** The initial reaction of pancreatic lipase was linear (not curvilinear or sigmoidal) in the presence of inhibitor, which means the reaction of pancreatic with substrate and inhibitor reached equilibrium very rapidly.

(a)


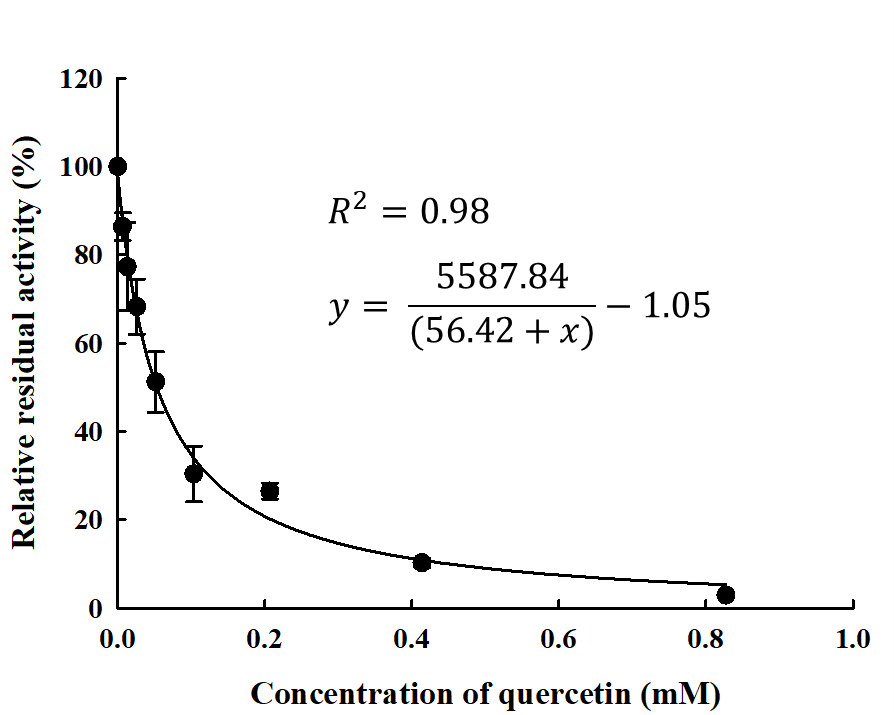


(b)


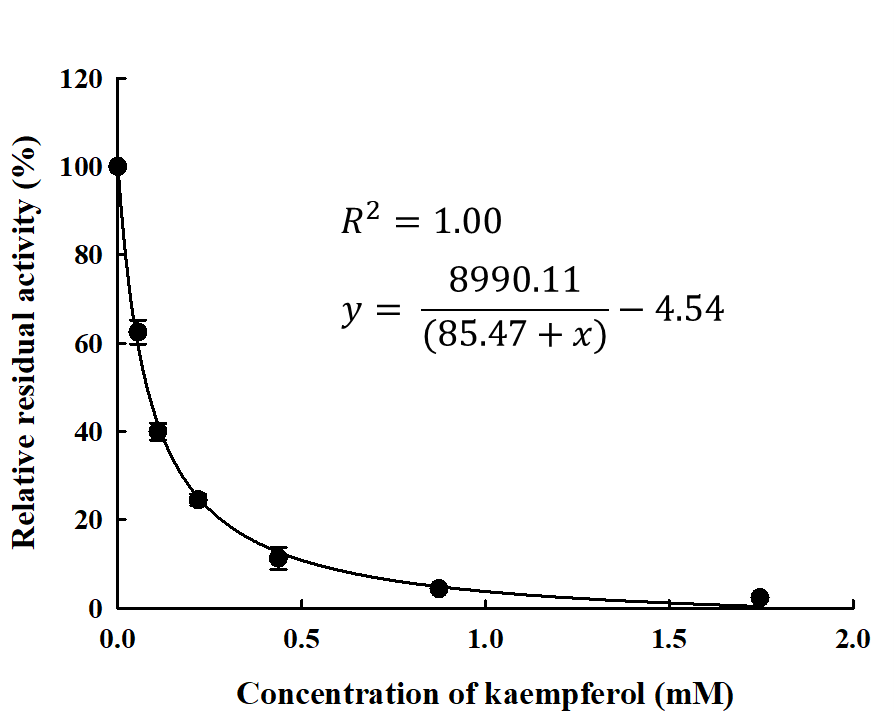


(c)


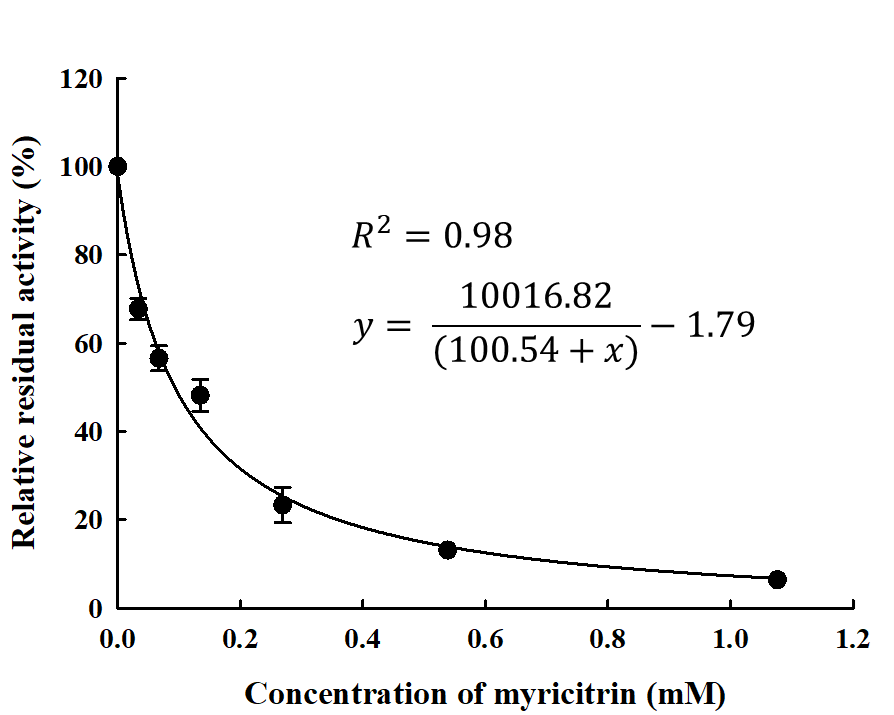


(d)


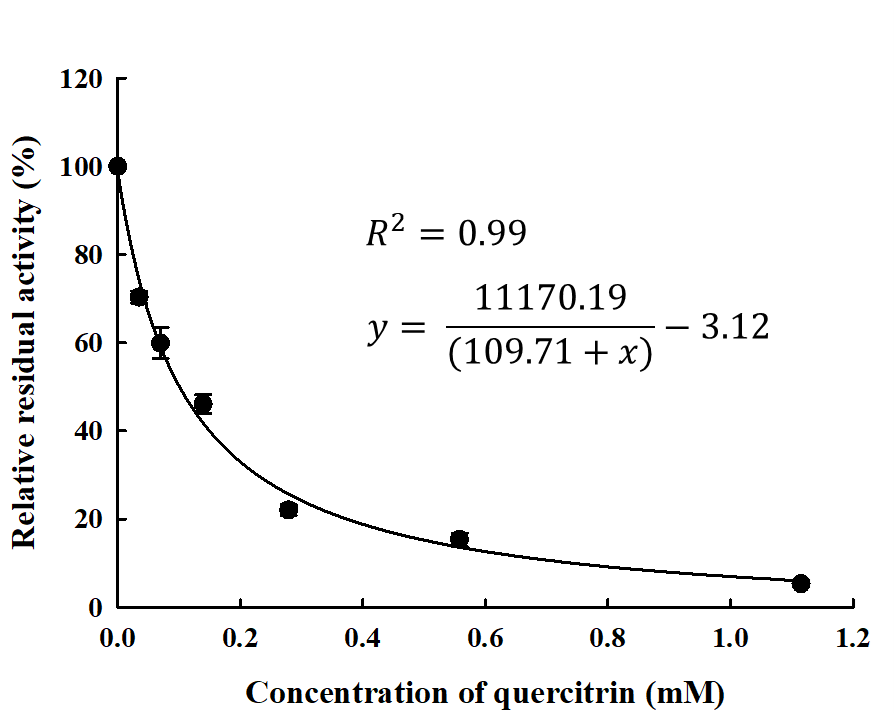


(e)


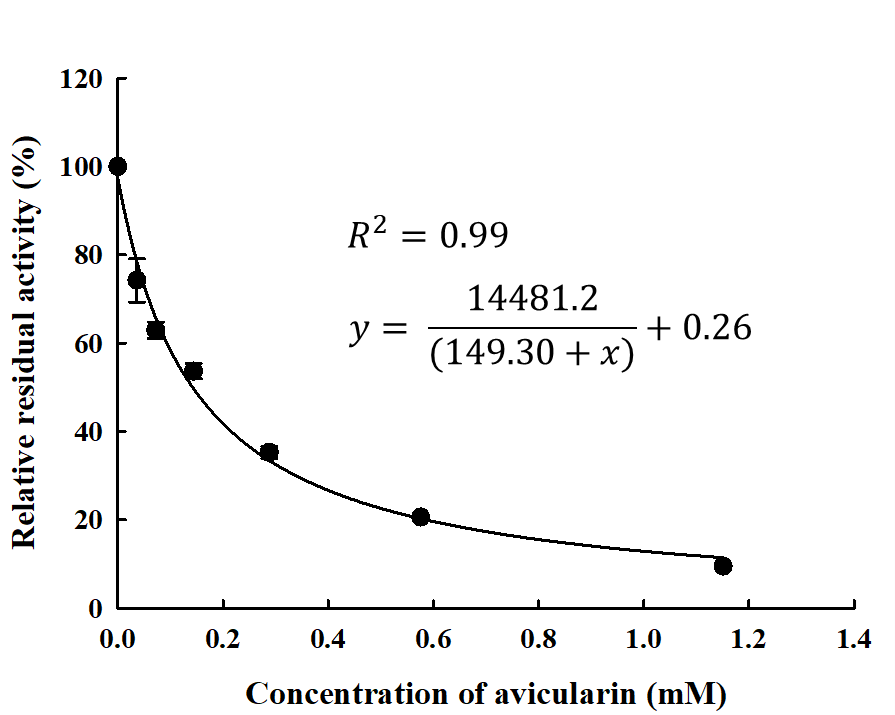


Figure S2. **Relative residual activity of pancreatic lipase in the presence of the major compounds and flavonol aglycones at different concentrations.** (A) Quercetin. (B) Kaempferol. (C) Myricitrin. (D) Quercitrin. (E) Avicularin. The half-maximal inhibitory concentrations of each compound were calculated from the equations.

(a)


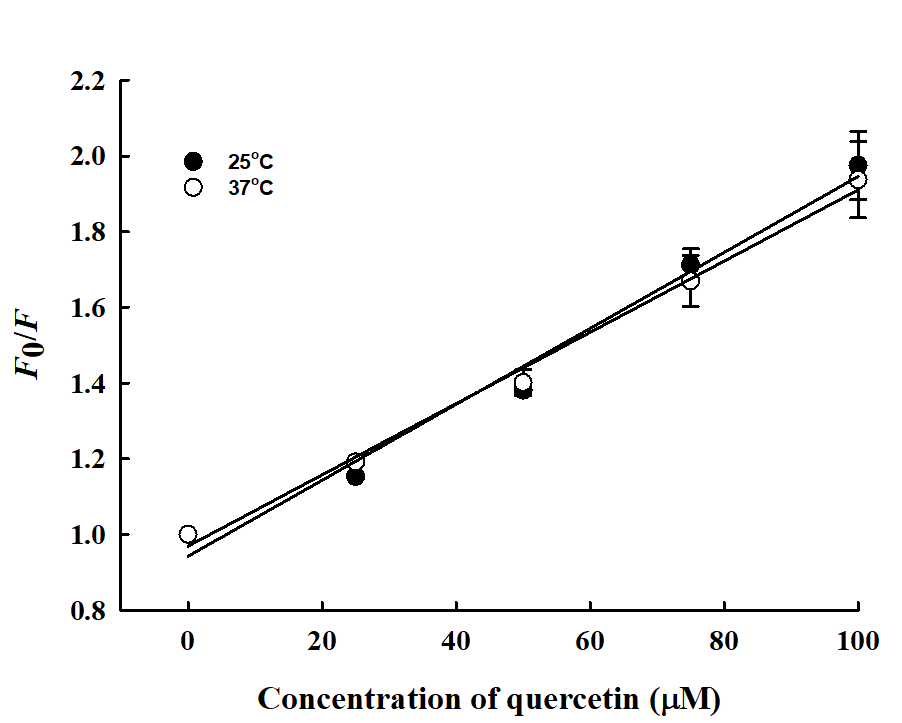


(b)


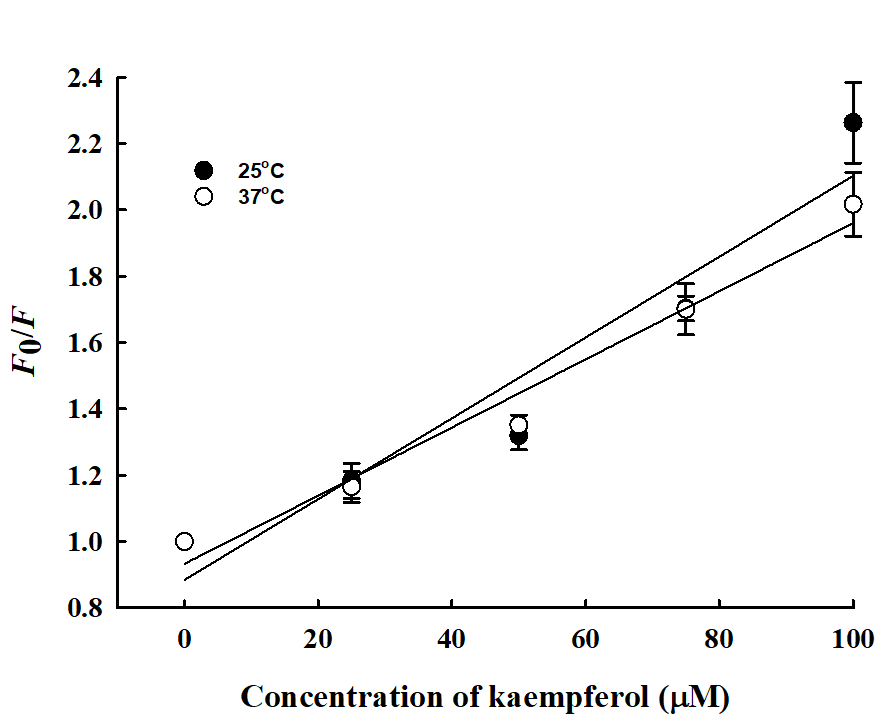


(c)


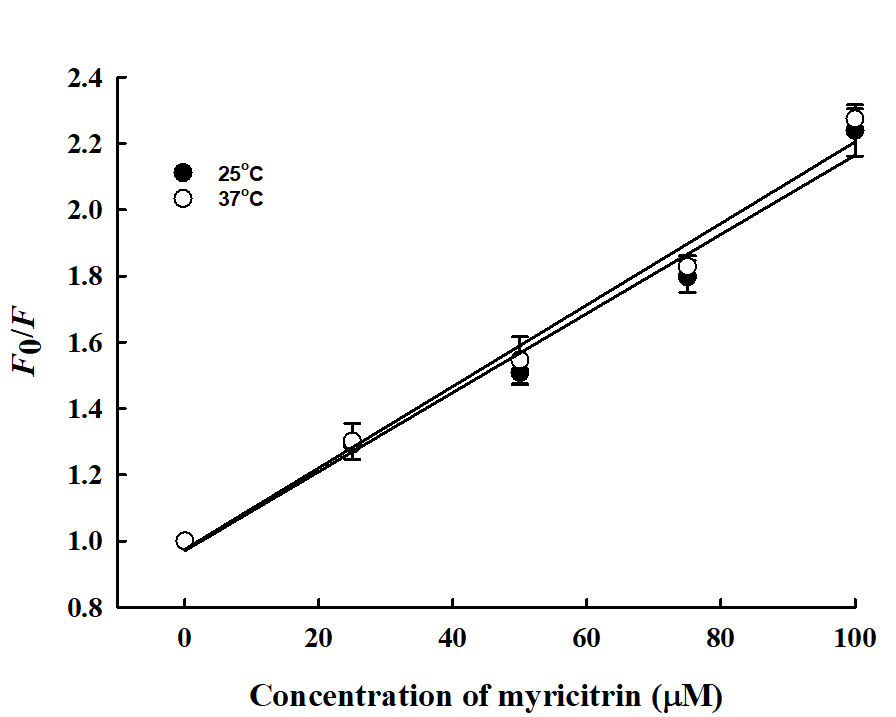


(d)


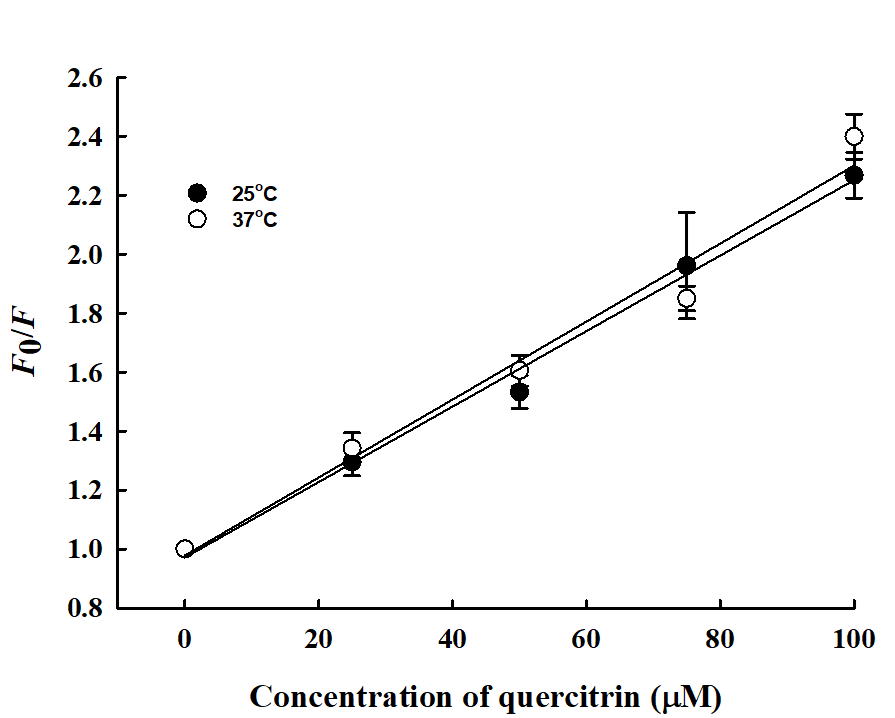


(e)


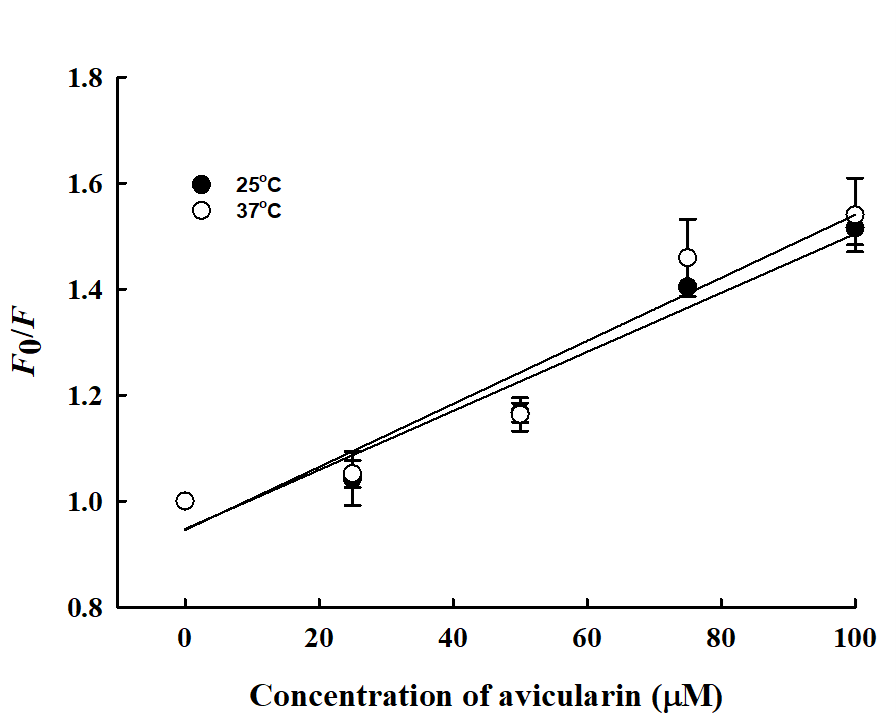


Figure S3. **Stern-Volmer plots of fluorescence quenching of pancreatic lipase in the presence of the major compounds and flavonol aglycones at 25^o^C and 37^o^C. (**A) Quercetin. (B) Kaempferol. (C) Myricitrin. (D) Quercitrin. (E) Avicularin. *F*_0_ and *F* is the fluorescence intensity in the absence and presence of quencher, respectively. *K*_SV_ values were obtained from the slopes of each plot.

(a)


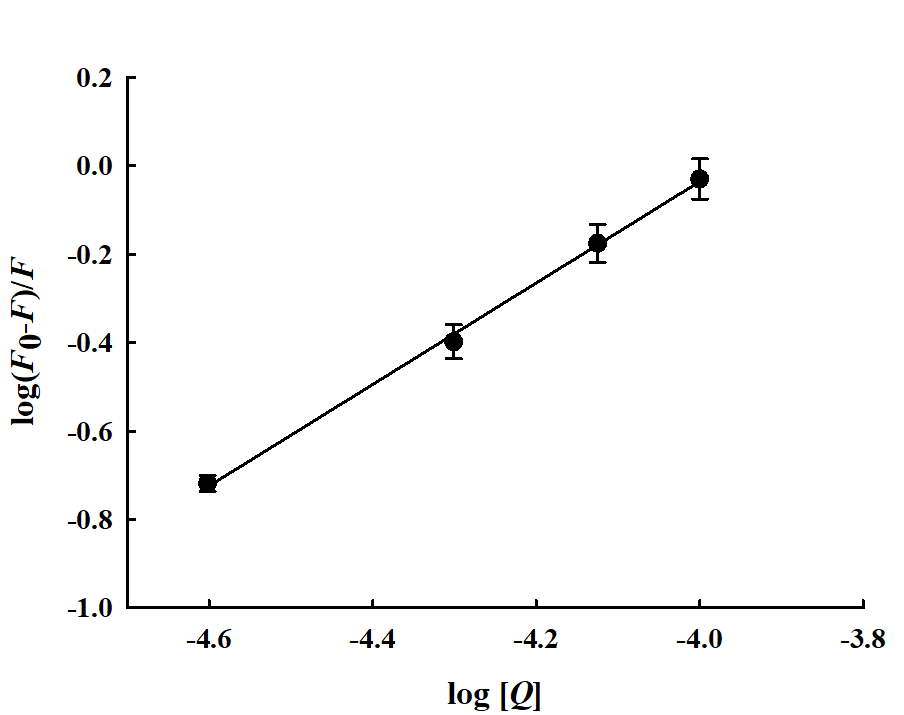


(b)


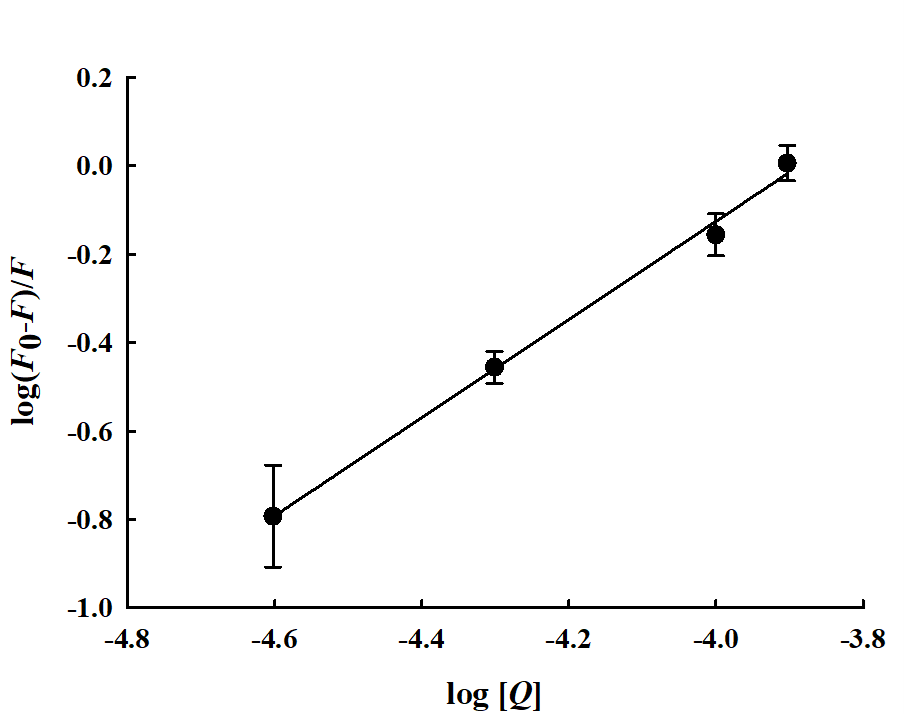


(c)


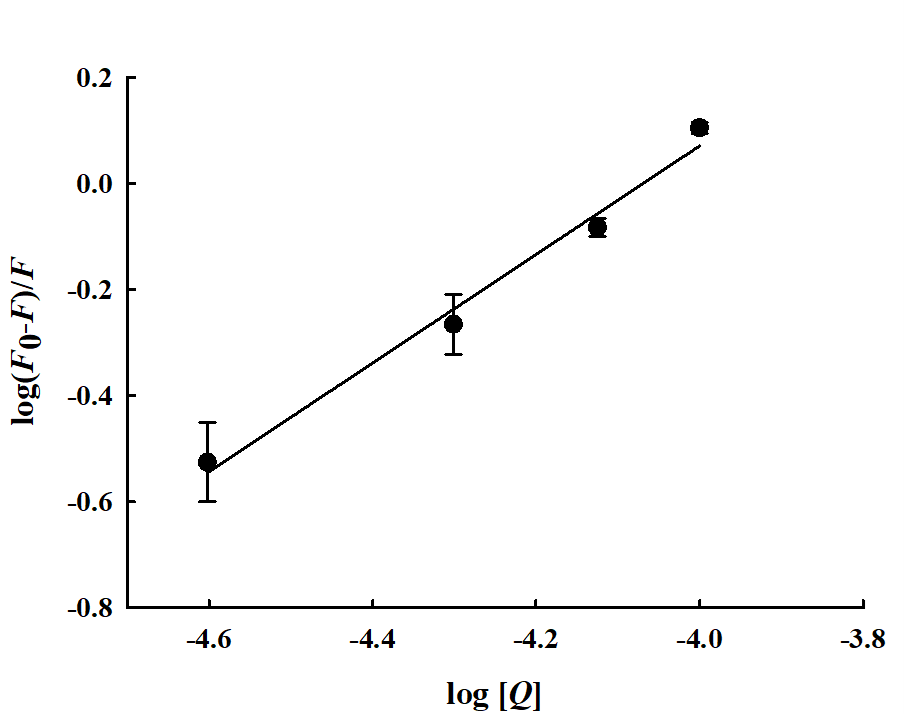


(d)


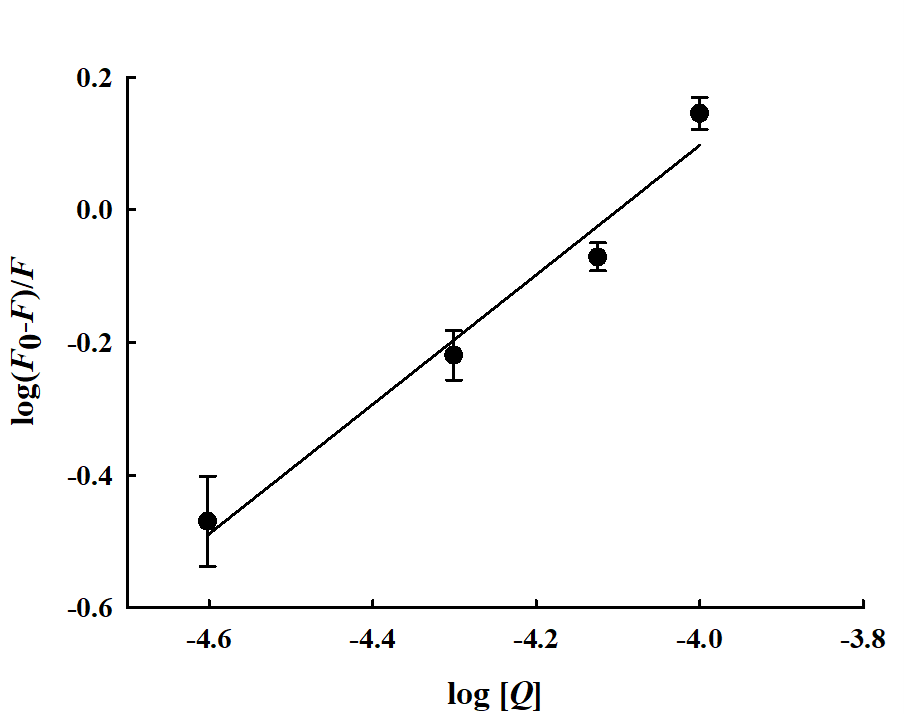


(e)


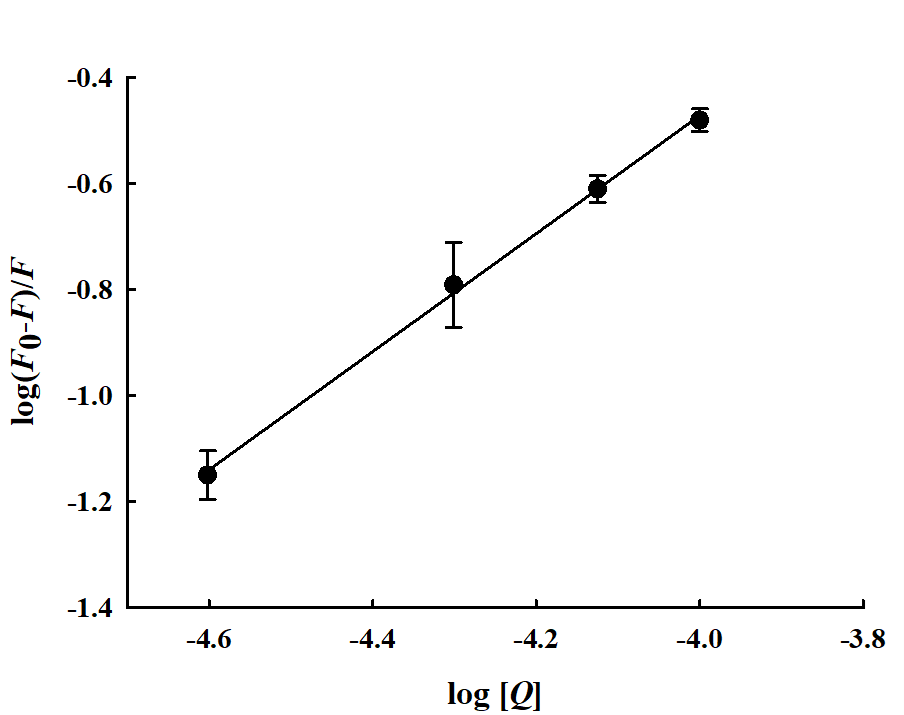


Figure S4. **Double-logarithmic plots of fluorescence quenching of pancreatic lipase in the presence of the major compounds and flavonol aglycones at 37^o^C. (**A) Quercetin. (B) Kaempferol. (C) Myricitrin. (D) Quercitrin. (E) Avicularin. *F*_0_ and *F* is the fluorescence intensity in the absence and presence of quencher, respectively. [*Q*] is the concentration of the compounds. The binding constant (*K*_A_) and number of binding sites (*n*) were obtained from each plot.


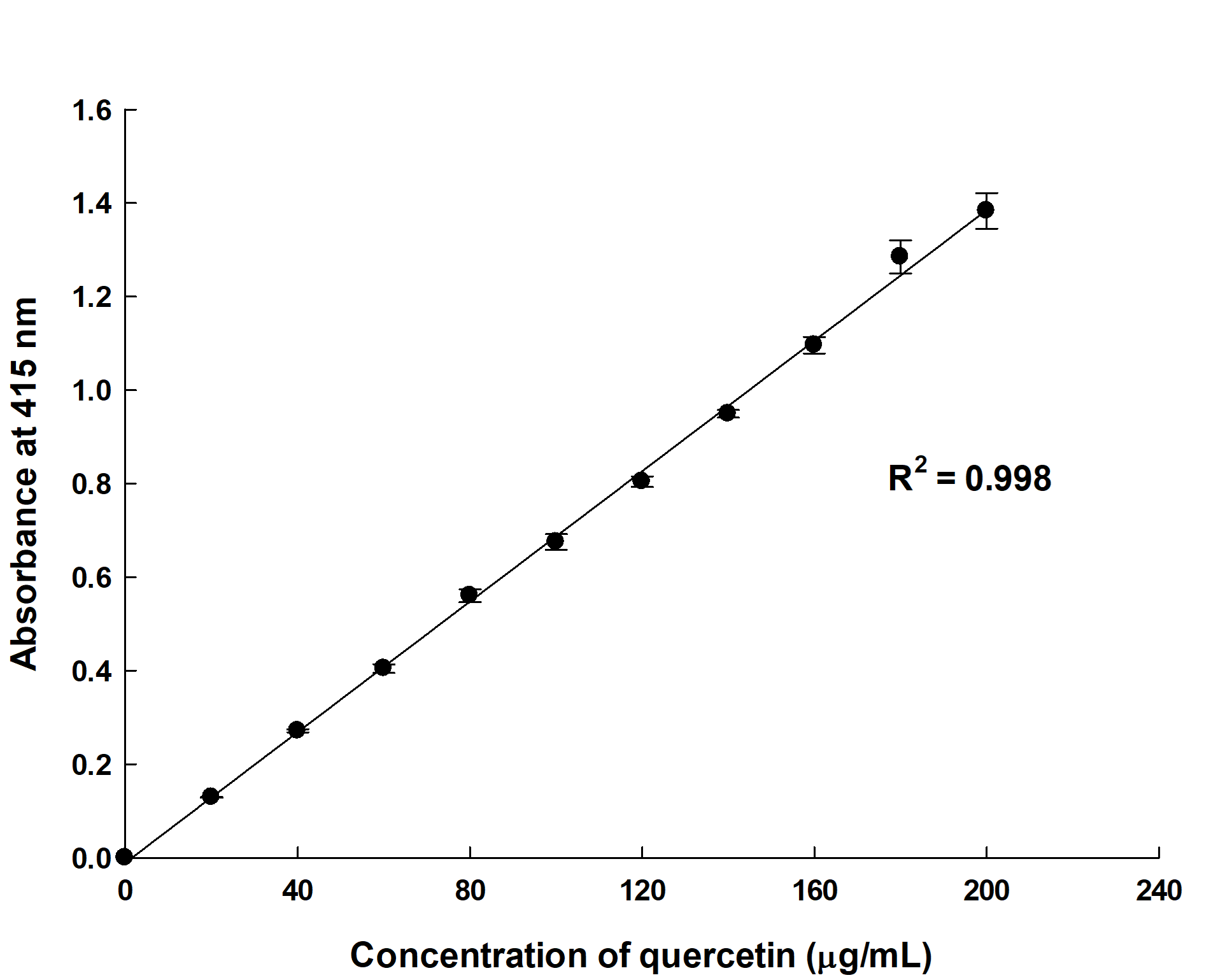


Figure S5. **Standard curve of quercetin for determination of total flavonoid content in *Polygonum aviculare* L. crude extract and its ethyl acetate fraction.**
